# Ethnic and Mouse Strain Differences in Central Corneal Thickness and Association with Pigmentation Phenotype

**DOI:** 10.1371/journal.pone.0022103

**Published:** 2011-08-10

**Authors:** David P. Dimasi, Alex W. Hewitt, Kenneth Kagame, Sam Ruvama, Ludovica Tindyebwa, Bastien Llamas, Kirsty A. Kirk, Paul Mitchell, Kathryn P. Burdon, Jamie E. Craig

**Affiliations:** 1 Department of Ophthalmology, Flinders University, Adelaide, South Australia, Australia; 2 Centre for Eye Research Australia, University of Melbourne, Royal Victorian Eye and Ear Hospital, Melbourne, Victoria, Australia; 3 Ruharo Eye Centre, Mbarara Municipality, Mbarara, Uganda; 4 School of Earth and Environmental Sciences, University of Adelaide, Adelaide, South Australia, Australia; 5 Centre for Vision Research, Department of Ophthalmology and Westmead Millennium Institute, University of Sydney, Westmead, New South Wales, Australia; Johns Hopkins University, United States of America

## Abstract

The cornea is a transparent structure that permits the refraction of light into the eye. Evidence from a range of studies indicates that central corneal thickness (CCT) is strongly genetically determined. Support for a genetic component comes from data showing significant variation in CCT between different human ethnic groups. Interestingly, these studies also appear to show that skin pigmentation may influence CCT. To validate these observations, we undertook the first analysis of CCT in an oculocutaneous albinism (OCA) and Ugandan cohort, populations with distinct skin pigmentation phenotypes. There was a significant difference in the mean CCT of the OCA, Ugandan and Australian-Caucasian cohorts (Ugandan: 517.3±37 µm; Caucasian: 539.7±32.8 µm, OCA: 563.3±37.2 µm; *p*<0.001). A meta-analysis of 53 studies investigating the CCT of different ethnic groups was then performed and demonstrated that darker skin pigmentation is associated with a thinner CCT (*p*<0.001). To further verify these observations, we measured CCT in 13 different inbred mouse strains and found a significant difference between the albino and pigmented strains (*p* = 0.008). Specific mutations within the melanin synthesis pathway were then investigated in mice for an association with CCT. Significant differences between mutant and wild type strains were seen with the nonagouti (*p*<0.001), myosin VA (*p*<0.001), tyrosinase (*p* = 0.025) and tyrosinase related protein (*p* = 0.001) genes. These findings provide support for our hypothesis that pigmentation is associated with CCT and identifies pigment-related genes as candidates for developmental determination of a non-pigmented structure.

## Introduction

The cornea is a transparent, avascular tissue that covers the anterior surface of the eye. The cornea plays a fundamental role in refracting light into the eye, as well as acting as a protective barrier against the extra-ocular environment and pathogens. Interest in central corneal thickness (CCT) has become widespread in the recent literature as its association with several ocular and non-ocular conditions becomes recognized. Most notably, CCT has been identified as a risk factor for the potentially blinding disease open-angle glaucoma [1]–[3], but abnormal measurements have also been shown to manifest in anirida [4], [5], Ehlers-Danlos syndrome [6]–[8], Marfan syndrome [9], [10] and osteogenesis imperfecta [11], [12]. In order to further understand the clinical relevance of this trait, the mechanisms underlying normal CCT variation need to be elucidated. Within the general population, CCT is a normally distributed quantitative trait, with a large meta-analysis conducted by Doughty and Zaman concluding that the mean of 230 different datasets was 536±31 µm [13]. As there is scant evidence of any environmental factors influencing CCT, it is probable that genetic factors play an important role in the determination of this trait. Indeed, evidence from both twin and familial studies indicate that CCT is highly heritable [14]–[16] and recent studies have identified several genes associated with normal variation in CCT [17]–[21].

Further evidence of the genetic nature of CCT comes from numerous studies which have found that CCT varies between different ethnic populations. Comparison of measurements from a range of ethnic groups, including people of African descent, American Indians/Alaskan natives, Australian Aborigines, Caucasians, Hispanics and several Asian populations has provided clear evidence that ethnicity influences CCT [22]. Whilst comparing the results of different studies is difficult, several present similar findings. Caucasians for example, have been consistently associated with a thicker CCT than both their African and Australian Aboriginal counterparts. In the absence of any confirmed environmental factors, this data implies that population specific genetic variants could be responsible for the variation in CCT. Given the striking differences in skin pigmentation between these groups, it also follows that dark skin is associated with a lower CCT.

To further investigate the relationship between human skin pigmentation and CCT, we assessed CCT in two human populations that had never previously been investigated for this trait, a group of individuals with oculocutaneous albinism (OCA) and a cohort from Uganda. The distinct skin pigmentation phenotypes of the OCA and Ugandan groups allowed for analysis of the role of pigmentation on CCT. In addition, a meta-analysis of all published human CCT data was undertaken, which included the Ugandan and Caucasian cohorts assessed in this study. The purpose of the meta-analysis was to systematically investigate whether any association between CCT and skin pigmentation can be observed following examination of the literature. In order to validate the observations seen in human studies, we then examined CCT in 13 different inbred mouse strains. The pigmentation status of the mouse coat colours was varied, with seven albino, two black, two dilute brown and two agouti strains. This permitted a direct examination of whether the pigmentation phenotype is correlated with CCT. The potential association between skin pigmentation and a transparent tissue such as the cornea is an extremely novel finding and could potentially lead to the identification of genes involved in new developmental pathways.

## Methods

### Ethics statement

Ethics approval for the research conducted on human participants was obtained from the human research ethics committees of Flinders University/Southern Adelaide Health Service, Westmead Millennium Institute at the University of Sydney and the Mbarara University of Science and Technology (Application number 16/056). Ethics approvals for work conducted on the mice used in this study were granted by the Animal Welfare Committee of Flinders University (Application number 608/05). All ethics approvals were granted following written submissions. This research adhered to the tenets of the Declaration of Helsinki and informed consent was obtained from all participants.

### Measurement of CCT in human participants

CCT was measured either by a contact ultrasound pachymeter (Pachmate DGH55, DGH-KOI, Inc. Shermans Dale, PA, USA) or by a non-contact slit-lamp mounted Optical Low Coherence Reflectometry (OLCR) pachymeter (Haag-Streit, Switzerland). Prior to contact pachymetry topical anaesthetic drops (either 0.4% oxybuprocaine or 0.5% proxymetacaine) were administered to both eyes 1 minute before measurement. An average of twenty-five consecutive measurements were recorded in each eye, such that each recording had a standard deviation <5 µm. An individual's CCT was calculated as mean of both eye measurements. Ultrasound and OLCR were found to have excellent correlation (data not shown). A detailed ocular history and ocular examination was performed on all participants and people who had anterior segment disease or previous refractive surgery were excluded from the study.

Ugandan participants (n = 297) were recruited from the Ruharo Eye Centre located in the Mbarara Municipality. Caucasian subjects (n = 956) were recruited through the Blue Mountains Eye Study (BMES). The BMES is a population-based cohort study investigating the aetiology of common ocular diseases among suburban residents aged 49 years or older, living in the Blue Mountains region, west of Sydney, New South Wales, Australia. Participants were recruited during a recent survey performed in 2004 and the full recruitment methodology has been described previously [23]. People with OCA (n = 22) were recruited during a National Albinism Fellowship Conference. There were 15 confirmed cases of OCA 1A and one case of OCA 1B, whilst the clinical diagnosis was inconclusive on the remaining subjects. All OCA patients were of Australian Caucasian descent.

All statistical analyses were conducted using SPSS v18.0. Differences in age and sex distribution amongst the BMES, OCA and Ugandan cohorts were assessed using the one-way ANOVA and chi-square procedures respectively. A two-way analysis of covariance (ANCOVA) was used to assess if significant differences in mean CCT were evident between the BMES, OCA and Ugandan cohorts. The two-way ANCOVA procedure allows for control of covariates such as age and sex.

### Meta-analysis

**Figure 1:**
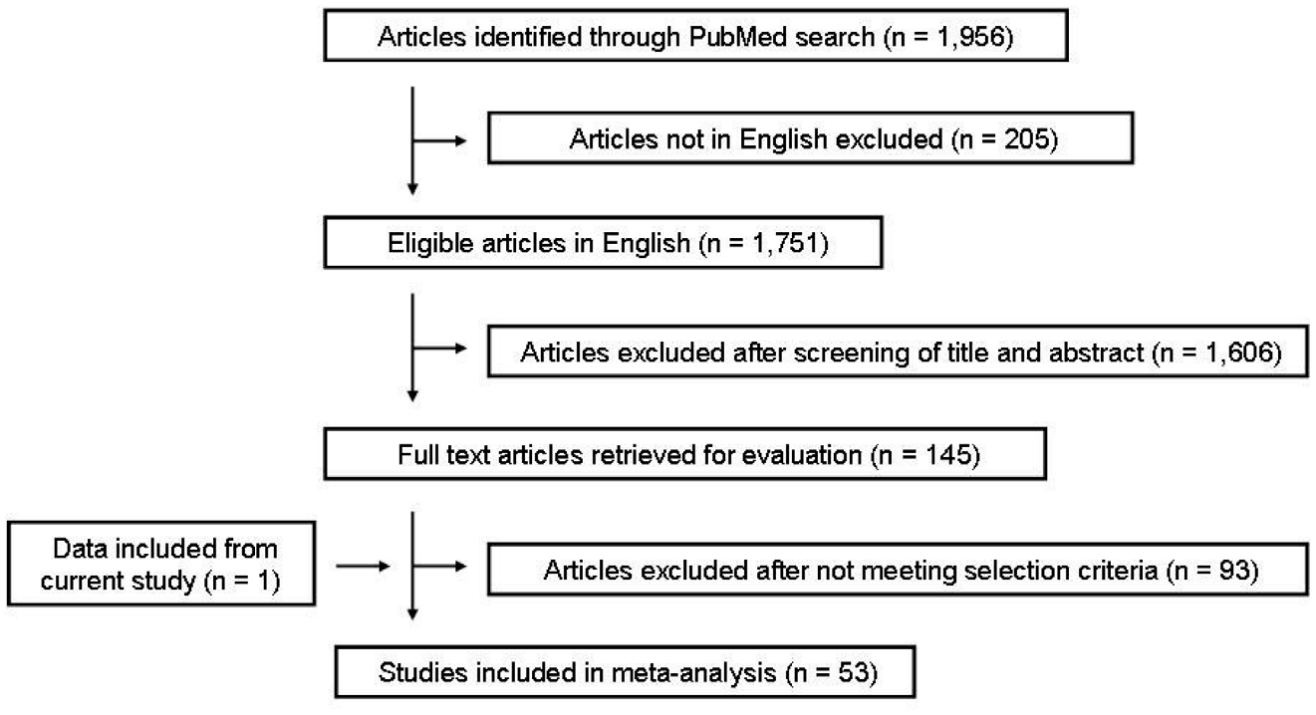
Flow chart detailing the selection process for articles included in the meta-analysis.

A systematic literature search was performed to identify all published studies that investigated central corneal thickness in human populations. The PubMed database (National Center for Biotechnology Information; NCBI) was explored in August 2010 by using the following keyword string: ‘central corneal thickness’. A total of 1,956 articles were identified in the PubMed database. Only articles in English were reviewed. Several criteria had to be met for an article to be included in the meta-analysis. To ensure consistency of data, measurement of CCT had to be performed by ultrasound pachymetry, as variation in CCT measurements has been demonstrated between different instruments [24]–[26]. As there is evidence to suggest that CCT decreases slightly with age [27]–[30], only studies involving adult populations (mean age or recruitment >18 years) were included. The study group had to comprise normal, healthy corneas and be predominately made up of normal eyes, although cohorts that contained a proportion of glaucomatous or ocular hypertensive eyes at normal population rates were permitted. An implicit statement regarding the ethnicity of the population being investigated had to be included in the article and the study group had to be ethnically homogeneous. Reporting of all necessary data such as cohort size, mean CCT values and variance was also required. There was no limit applied with regards to the date at which the article was published. The final number of articles that qualified for inclusion in the meta-analysis was 53, which included the addition of the Caucasian and Ugandan data from this study. A flow chart of the study selection process, including the number of articles excluded at each phase, can be seen in Figure 1.

**Figure 2:**
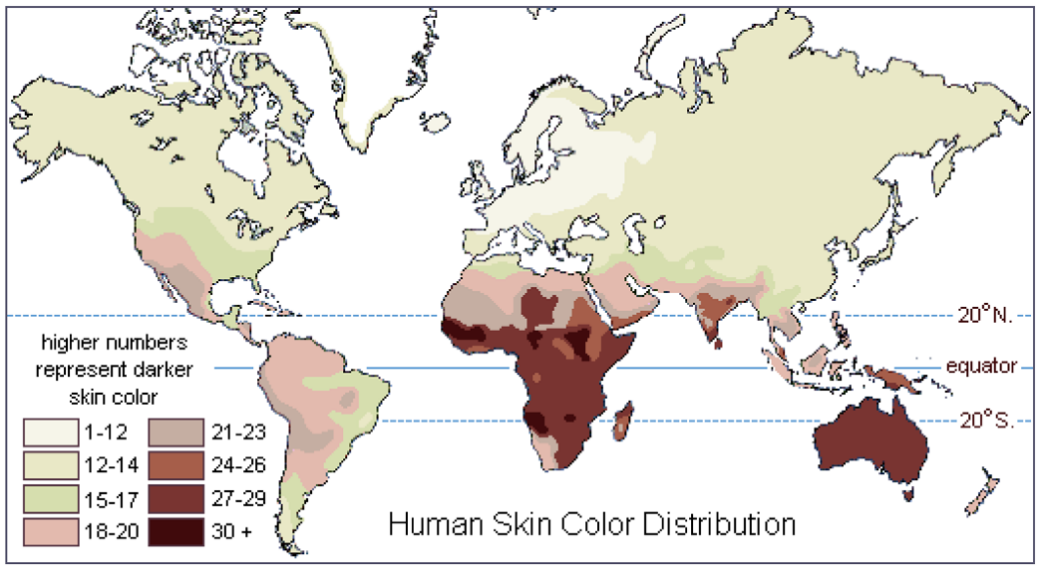
Global skin colour distribution of native populations. The colours on the map are based on the 36-tone chromatic scale devised by Austrian anthropologist Felix von Luschan to assess the unexposed skin of human populations. The higher numbers represent darker skin colour. Original data compiled by Biasutti 1941.

Articles that met the inclusion criteria stated above are shown in Table S1. The Caucasian and Ugandan data from this study was also included in the meta-analysis. Each study population was assigned to an ethnic group based on the reported ethnicity and geographical location of the recruitment. Geographical regions were based on definitions used by the United Nations Population Division (http://www.un.org/esa/population/). For African and Caucasian ethnic groups that were recruited outside of Africa and Europe respectively, these groups were designated ‘African Migrant’ and ‘Caucasian Migrant’. Each ethnic group was assigned to the ‘Light Skin’ or ‘Dark Skin’ cohorts based on the map of native skin colour distributions compiled by Biasutti in 1941 (Figure 2) [31]. The data in this map is based on the 36-tone chromatic scale devised by Austrian anthropologist Felix von Luschan to assess the unexposed skin of human populations. In general, pinkish-white skin corresponds to 1–12 on the scale; white 12–14; white-light brown 15–17; light brown 18–23; brown 24–26; dark brown 27 or above. It was decided that the ‘Light Skin’ cohort would consist of ethnic groups that had a skin tone between 1–17 on the von Luschan scale and the ‘Dark Skin’ cohort would consist of ethnic groups who were 18 or above. Therefore, the groups were assigned as follows: Light Skin; Caucasian European, Caucasian Migrant, East Asian. Dark Skin; Australian Aboriginal, African Native, African Migrant, Hispanic, South Asian, South East Asian. Even though the American Hispanic population can be quite heterogeneous in terms of ancestry, a large proportion are descendants of Central and Southern America and as such, were designated as having a skin tone of 18 or above.

The calculation of mean CCT and standard deviation values for each ethnic group was performed by compiling data from each study and weighting it according to the number of participants. This data was used in the calculation of mean CCT and standard deviation values for the ‘Light Skin’ and ‘Dark Skin’ groups, which was also weighted according to the number of participants. Comparison of the mean CCT values of the ‘Light Skin’ and ‘Dark Skin’ groups was undertaken using Student's *t* test in Microsoft Excel 2003 and statistical significance was accepted as *p*<0.05. As complete data on age and sex were not available from all studies, correction for these variables was not performed. This meta-analysis conformed to the Preferred Reporting Items for Systematic Reviews and Meta-Analyses (PRISMA) guidelines [32].

### Measurement of CCT in the mouse

For all strains, CCT was measured in adult female mice between 6–10 weeks of age as this minimised the potential effect sex and age may have on measurements. Mice were weighed then sedated with inhaled isoflurane before being anaesthetised by intraperitoneal injection of 75 mg/kg ketamine and 30 µg/kg metatomidine. Pupils of both eyes were dilated using tropicamide drops. Animals were placed on an improvised support structure mounted on the slit lamp to enable correct height adjustment with the OLCR (Haag-Streit, USA). This method for CCT measurement in animals has been verified previously [33]. Right eye measurements were taken unless there was an existing problem, such as corneal scarring, in which case the left eye was measured. In order to prevent the corneal surface from drying out, a 2 µl drop of saline solution was administered 30 seconds prior to taking the first measurement. Each reading consisted of 10 measurements with the highest and lowest measurements excluded. The instrument then presents the mean of 8 measurements with a standard deviation. At least 2 readings with a standard deviation of less than 2 µm taken within 3 minutes of the administration of the saline were required for inclusion. An overall mean measure was then calculated for each animal.

### Mouse strains and statistical analysis

Inbred mouse strains for this study were obtained from the following institutes: Adelaide University, Adelaide, Australia (AU); Animal Resource Centre, Perth, Australia (PARC); Canberra Hospital, Canberra, Australia (CH); Gilles Plains Animal Resource Centre, Adelaide, Australia (GPARC); The Jackson Laboratory, Bar Harbor, USA (JAX); Walter and Eliza Hall Institute, Melbourne, Australia (WEHI). The following strains of mice were included in this study (source of strain in parentheses): 129X1/SvJ (WEHI); A/J (JAX); AKR (PARC); BALB/c (GPARC); C3H/HeJ (JAX); CBA/CaH (AU); C57B1/KALWRIJ (PARC); C57BL/6J (JAX); DBA/1J (JAX); DBA/2J (JAX); FVB/NJ (PARC); NOD/Lt (CH); SJL/J (PARC). All animals were on the same diet and housed under identical conditions, which included a room temperature of 21°C, 50% humidity and a 12/12 hour light/dark cycle in the Flinders Medical Centre Animal Facility. The 13 inbred strains were classified as either albino or pigmented based on their coat colour: Albino; 129X1/SvJ, A/J, AKR, BALB/c, FVB/NJ, NOD/Lt, SJL/J. Pigmented; C3H/HeJ, CBA/CaH, C57B1/KALWRIJ, C57BL/6J, DBA/1J, DBA/2J. The genotype of each strain (excluding C57B1/KALWRIJ, as no genotype information was available) at five known mouse coat colour genes was determined from information found on the Jackson Laboratories website (http://www.jax.org/). The five genes selected were the mouse homologue of *ASIP* known as nonagouti (*a*), myosin VA (*Myo5a*), *Oca2* (also known as the *p* gene), tyrosinase (*Tyr*) and tyrosinase related protein (*Tyrp1*). The genotype of each mouse strain at each gene is given in Table 1. All the mutations investigated are recessive and the observed phenotype is dependent on the combination with other alleles. In general each mutation results in the following pigment phenotypes; *a -* black coat, A^w^ - light-bellied agouti [34]; *Myo5a^d^* - dilution of hair pigmentation [35]; *Oca2^P^* - reduction in coat and eye pigmentation [36]; *Tyr^c^ -* albino [37]; *Tyrp1^b^* - brown coat [38].

**Table 1:** Genotype of each mouse strain for five pigment associated genes.

		Allele at coat colour loci				
Strain	Coat Colour	*a*	*Myo5a*	*Oca2*	*Tyr*	*Tyrp1*
129X1/SvJ	Albino	A^w^	+	Oca2^P^	Tyr^c^	+
A/J	Albino	a	+	+	Tyr^c^	Tyrp1^b^
AKR	Albino	a	+	+	Tyr^c^	+
BALB/c	Albino	+	+	+	Tyr^c^	Tyrp1^b^
C3H/HeJ	Agouti	+	+	+	+	+
CBA/CaH	Agouti	+	+	+	+	+
C57BL/6J	Black	a	+	+	+	+
DBA/1J	Dilute Brown	a	Myo5a^d^	+	+	Tyrp1^b^
DBA/2J	Dilute Brown	a	Myo5a^d^	+	+	Tyrp1^b^
FVB/NJ	Albino	+	+	+	Tyr^c^	+
NOD/Lt	Albino	+	+	NA	Tyr^c^	+
SJL/J	Albino	+	+	Oca2^P^	Tyr^c^	+

For each gene, the wild-type allele is designated by the ‘+’ symbol. The ‘A^w^’ allele of the *a* gene carried by the 129X1/SvJ strain is a distinct variant that has been classified as wild-type for this study. No genotype information was available for the C57BL/KALWRIJ strain. NA indicates an unavailable genotype.

Correlation between weight, age and CCT was assessed using the Pearson correlation coefficient. General strain differences were tested using a Kruskal-Wallis test. The mean CCT of pigmented animals was compared to that of albino animals using a Mann-Whitney U test. Statistical significance was defined as *p*<0.05.

## Results

### Human study

**Figure 3:**
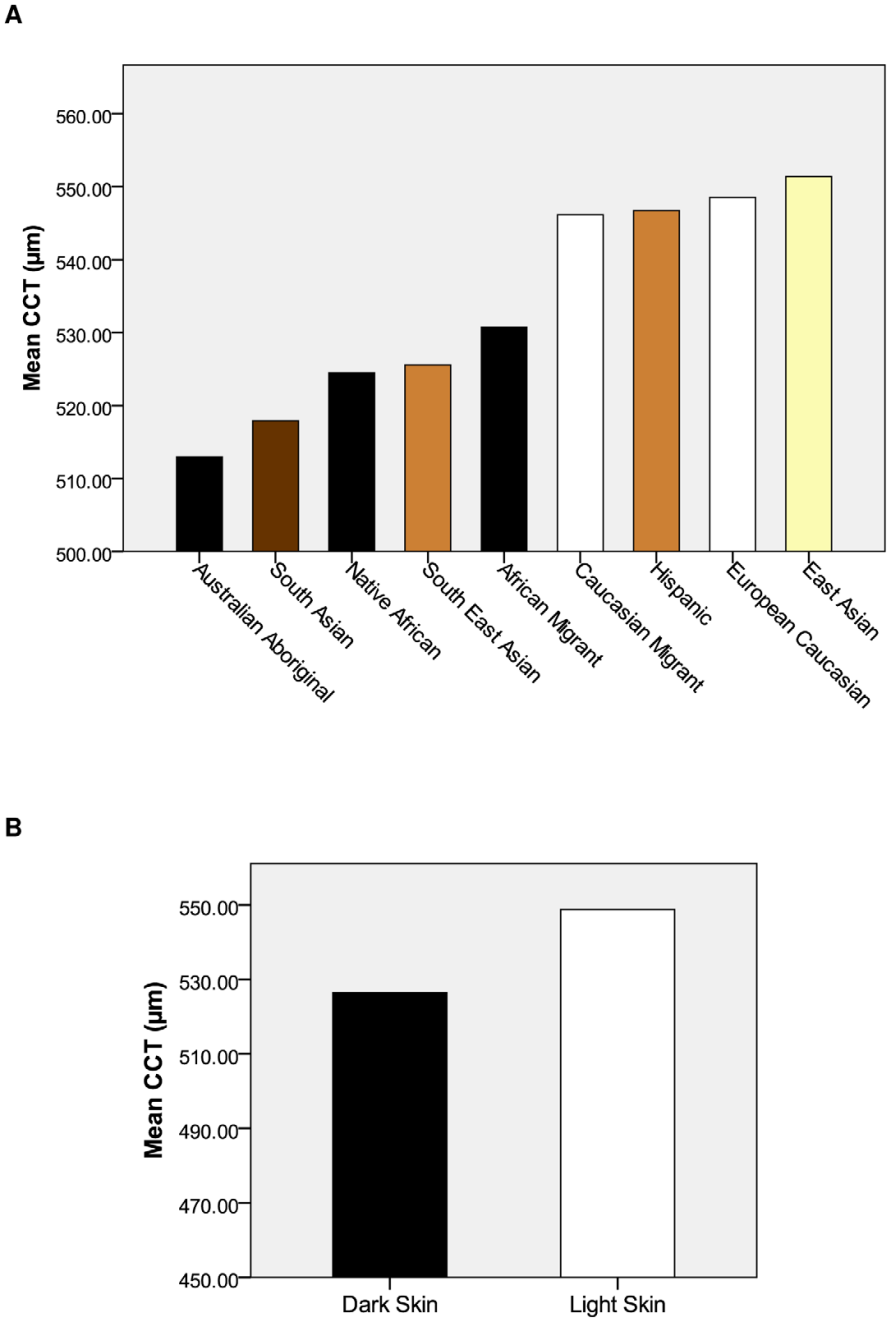
Graphical representation of the human CCT meta-analysis results. (**A**) Mean CCT of each ethnic group. Colours indicate tone of skin pigmentation according to the chart devised by Biasutti, 1941 (see Figure 1) (**B**) Mean CCT of the Dark Skin (524.6±33.6 µm, n = 16,472) and Light Skin (548.4±34.1 µm, n = 14,152) groups based on the skin colour of the ethnic groups in Figure 1A. There was a significant difference between the groups (*p*<0.001).

Data from the human cohorts is shown in Table 2. Age of participants in the BMES ranged from 60 to 95 years, from 5 to 65 years in the OCA and from 5 to 90 in the Ugandan cohorts. There were 62 (6.5%) confirmed cases of open-angle glaucoma in the BMES. There was a 46 µm difference in mean CCT between the thickest (OCA) and thinnest (Ugandan) cohorts. After adjusting for age and gender, there was a significant difference in the mean CCT between each of the three cohorts (*p*<0.001) (Table 2). Results from the meta-analysis of human CCT data are shown in Table 3 (also see Figure 3A). In all, 53 studies were included in the meta-analysis with data from 76 different ethnic groups. Across all ethnic groups there was a broad range of measurements, with a 38.4 µm difference in mean CCT between the thinnest (Australian Aboriginals) and thickest (East Asian) groups. Following segregation of the ethnic groups into two groups based on skin pigmentation, the mean CCT measurements were as follows: Dark Skin = 524.6±33.6 µm (n = 16,472); Light Skin = 548.4±34.1 µm (n = 14,152) (*p*<0.001) (Figure 3B).

**Table 2:** Characteristics of human cohorts.

	BMES	OCA	Ugandan	*p* value
N	956	22	297	
Mean Age (years)	73.8	35.4	40.8	**<0.001**
Gender (% Female)	59.9	72.7	46.5	**<0.001**
Mean CCT ± SD (µm)	539.7±32.8	563.3±37.2	517.3±37	**<0.001**

The number of participants (N), mean age in years, percentage of females and mean CCT is shown for each cohort included in the study. Values in bold are considered significant at the *p*<0.05 level. BMES = Blue Mountains Eye Study, OCA = oculocutaneous albinism, SD = standard deviation.

**Table 3:** Mean CCT for each ethnic group assessed in meta-analysis.

Ethnicity	Number of Studies	Number of Participants	Mean CCT ± SD (µm)
Australian Aboriginal	2	280	513±31.5
South Asian	6	8437	517.9±33.2
Native African	6	1320	524.5±35.6
South East Asian	4	2459	525.6±32.4
African Migrant	11	1905	530.8±35.8
Caucasian Migrant	16	5040	546.2±34.2
Hispanic	5	2071	546.7±33.7
European Caucasian	9	5588	548.6±34.5
East Asian	13	3524	551.4±33.5

The cumulative number of studies and total number of participants from these studies in each ethnic group is given. A mean CCT and standard deviation (SD) was calculated for each ethnic group with each study weighted according to size.

### Mouse study

**Figure 4:**
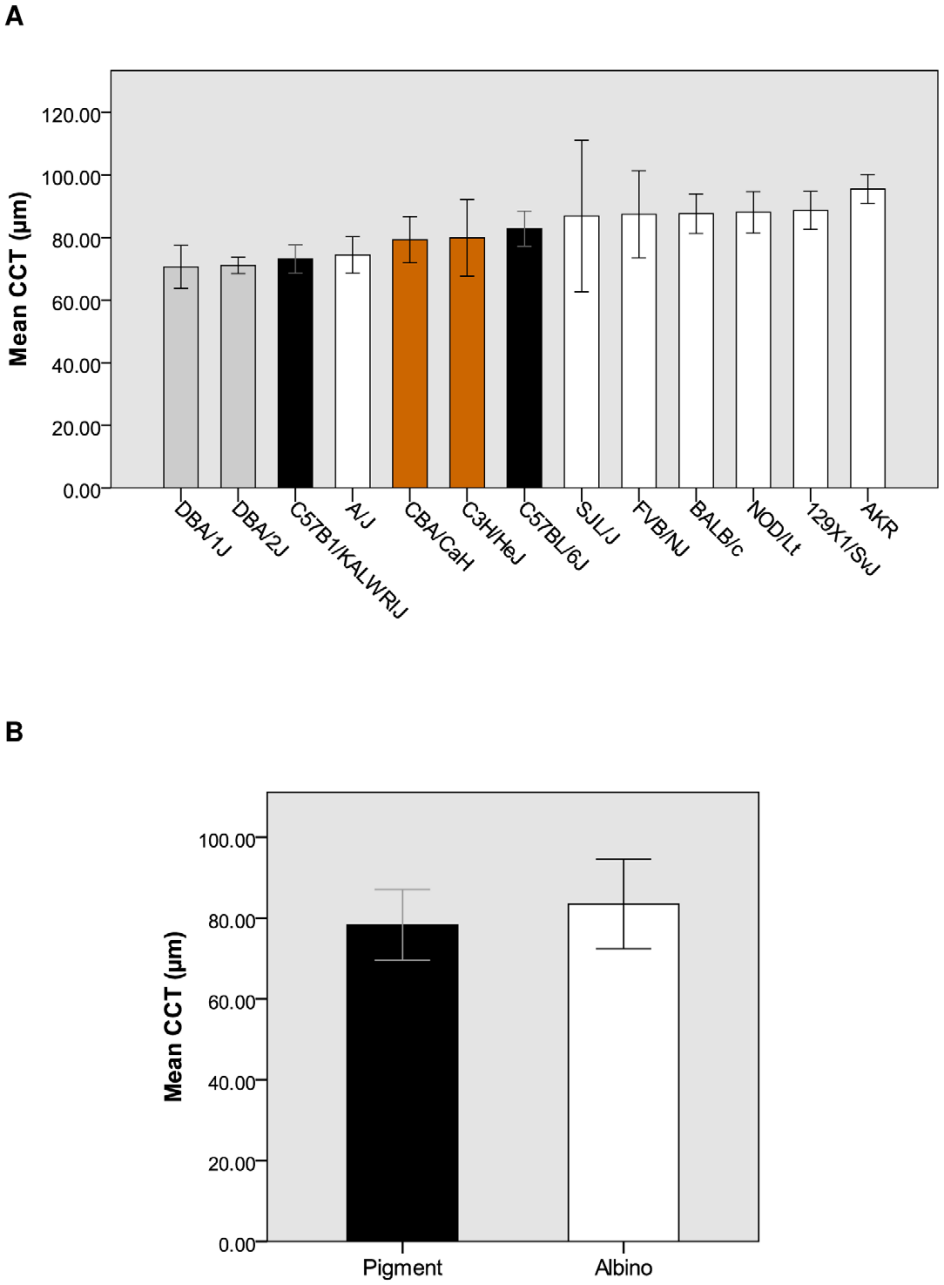
Graphical representation of CCT measurements conducted on the inbred mouse strains. (**A**) Mean CCT and coat colour of the each individual strain. There was a significant difference in mean CCT of each strain (*p*<0.001). The colours of the bars represent the coat pigmentation of the animals. Error bars indicate standard deviation. (**B**) Mean CCT of the Pigment (78.3±8.8 µm, n = 53) and Albino (83.5±11.1 µm, n = 79) groups based on the coat colour of the animals in Figure 3A. Error bars indicate standard deviation. There was a significant difference between the groups (*p* = 0.008).

Mean CCT readings from the 13 inbred mouse strains are displayed in Table 4 (also see Figure 4A). Across all strains there was a broad range of measurements, with a 24.9 µm difference in mean CCT between the thinnest (DBA/1J) and thickest (AKR) strains. There was a significant difference between the CCT measurements across all strains (*p*<0.001). There was no correlation between weight (*p* = 0.297) or age (*p* = 0.426) of the mice and CCT. Following segregation of the strains into two groups based on coat pigmentation, the mean CCT measurements were as follows: Pigmented = 78.3±8.8 µm (n = 53); Albino = 83.5±11.1 µm (n = 79) (*p* = 0.008) (Figure 4B). We also analysed the data based on the genotype status of five pigment associated mouse genes. All mice were divided into groups based on whether they were mutant or wild type at the particular locus and then assessed for any differences in mean CCT (Table 5). Significant differences were seen between mice carrying the wild type and mutant alleles of *a*, *Myo5a*, *Oca2, Tyr* and *Tyrp*. There was no significant difference in CCT in mice carrying the wild type and mutant alleles of *Oca2*.

**Table 4:** Coat colour and mean CCT of each inbred mouse strain.

Strain	Coat Colour	Number of Animals	Mean CCT ± SD (µm)
DBA/1J	Dilute Brown	6	70.6±6.9
DBA/2J	Dilute Brown	5	71.1±2.6
C57B1/KALWRIJ	Black	5	73.6±4.6
A/J	Albino	28	74.4±5.8
CBA/CaH	Agouti	6	79.3±7.4
C3H/HeJ	Agouti	12	79.9±12.3
C57BL/6J	Black	19	82.8±5.6
SJL/J	Albino	5	86.8±24.2
FVB/NJ	Albino	6	87.4±13.9
BALB/c	Albino	24	87.6±6.3
NOD/Lt	Albino	6	87.9±6.6
129X1/SvJ	Albino	4	88.6±6.1
AKR	Albino	6	95.5±4.6

SD = standard deviation.

**Table 5:** Mean CCT values for each genotype of the five pigment associated genes.

	Wild Type		Mutant		
Gene	Number	Mean CCT ± SD (µm)	Number	Mean CCT ± SD (µm)	*p* value
*a*	63	85.3±10.9	64	78.2±9.0	**<0.001**
*Myo5a*	116	82.8±10.3	11	70.8±5.1	**<0.001**
*Oca2*	112	81.0±9.8	9	87.6±17.6	0.121
*Tyr*	48	78.9±9.0	79	83.5±11.1	**0.025**
*Tyrp1*	64	84.7±11.0	63	78.8±9.2	**0.001**

The number of animals and mean CCT for each genotype of the five pigment associated genes is shown in the table. Values in bold are considered significant at the *p*<0.05 level. SD = standard deviation.

## Discussion

A healthy cornea is essential for normal vision, as it serves a number of roles that are vital to maintaining the structure and function of the eye. One of the primary roles of the cornea is to refract light into the eye, a property that is primarily due to the transparent nature of the tissue. Another important attribute of the cornea is the CCT, a normally distributed trait that requires assessment for several ocular disorders and is also associated with several non-ocular conditions. There is sufficient evidence from hereditary and genetic studies to demonstrate that normal variation in CCT is highly genetically determined and this is supported by the significant differences in CCT between distinct ethnic groups. Further analysis of the data from human ethnic groups reveals the intriguing observation that skin pigmentation appears to be associated with CCT, with dark skin populations such as African Americans and Australian Aborigines consistently exhibiting thinner CCT measurements than fairer skinned Caucasians [22]. Our research was designed to investigate this observation by assembling data from both human and mouse studies, including the first reported measurements of CCT in OCA and Ugandan populations. The outcomes from this study demonstrate that the thickness of the cornea is intimately related to the degree of skin or coat pigmentation.

In accordance with findings from other African and dark skin populations, the Ugandan cohort investigated in this study had a mean CCT that was significantly lower than our Australian-Caucasian cohort. In comparison to other ethnic groups, the Ugandan mean CCT of 517.3 µm was considerably thin and was the lowest measurement found in any African study performed using ultrasound (see Table S1). Only three studies performed with ultrasound have observed a lower CCT than that found in the Ugandans, with two of these from Australian Aboriginal cohorts and the other from an Indian population [39]–[41]. Consequently, the meta-analysis revealed that Australian Aboriginals have the lowest CCT measurements of any ethnic group, although the smaller number of participants assessed in this population suggests that further investigation is required to confirm this finding. The ethnic group with the second lowest CCT measurements as determined by the meta-analysis were the South Asians, which comprised predominately of Indians, followed by African natives, South East Asians and African migrants, indicating that the populations with the darkest skin pigmentation also had the lowest CCT measurements. When all the ethnic groups were segregated into ‘Light Skin’ and ‘Dark Skin’ cohorts, the ‘Dark Skin’ group had a significantly thinner CCT. This result was in support of the hypothesis that darker skin pigmentation is associated with a thinner CCT.

The data from the OCA patients, who were collectively found to have a significantly thicker CCT than our Australian-Caucasian population, supports our hypothesis that skin pigmentation is associated with CCT. OCA is a group of disorders characterised by congenital hypopigmentation of the skin, hair and eyes. Ocular abnormalities are also a major clinical sign of OCA and include nystagmus, foveal hypoplasia, colour vision impairment, reduced visual acuity and misrouting of the optic nerve fibres. Four different forms of OCA have been described, each presenting with varying degrees of skin and hair pigmentation. The underlying genetic defect is also unique to each form of OCA, with mutations in *TYR, P, TYRP1* and *SLC45A2* associated with OCA types 1–4 respectively [42]. Despite some genetic and phenotypic variability, all forms of OCA present with similar ocular abnormalities. Whilst not a distinct ethnic group, measurement of CCT in the OCA patients made for an interesting comparison. In terms of their skin phenotype, OCA patients have little or no skin pigmentation and thus sit at the opposite end of the spectrum when compared to African or Australian Aboriginal groups. The thicker CCT measurements in the OCA group are again consistent with our pigmentation hypothesis, as they are significantly thicker than all races of people with darker skin, including other Caucasians. This data from the OCA cohort may also offer insights into the genetic mechanisms that influence CCT in humans. The majority of patients in this study presented with OCA1, which results from mutations in *TYR* [42], [43]. Whilst the exact mutation is unknown in these patients, the results do identify *TYR* as a candidate gene for future research of CCT genetics.

Data showing an association between skin pigmentation and CCT in humans is supported by observations in the mouse. Measurements from the 13 inbred mouse strains used in this study definitively showed that CCT is influenced by pigmentary differences. A highly significant association was observed when comparing measurements across all strains (*p*<0.001) and there was no correlation with weight and CCT, as these were eliminated as potential covariates. Given our ability to control for variables such as the type of pachymeter, pachymeter operator and environmental conditions, it is highly probable that the genetic diversity between the strains is responsible for the differences in CCT. This data is consistent with the findings of Lively *et al*, where a significant difference in CCT was seen across 17 strains of mice [44]. Similarly, Henriksson *et al* demonstrated variation in CCT across three inbred mouse strains [45]. While comparisons can be made within studies, differences in measurement methodology make it difficult to compare the CCT readings across these studies however, with Lively *et al* employing ultrasound pachymetry, Henriksson *et al* utilising histology and our study employing OLCR. For example, we obtained a mean CCT measurement for BALB/c mice of 87.6±6.3 µm, whilst Henriksson *et al* found a mean of 134.2±12.9 µm for the same strain. Whilst the small size of the mouse eye makes accurate measurement of CCT problematic, previous research has confirmed the utility of using OLCR for this purpose [17], [33]. Further evidence that strain differences in CCT are genetically determined comes from a recent linkage study that identified a quantitative trait locus for CCT on mouse chromosome 7 [46]. If the mouse data is extrapolated to humans, then it would suggest that genetic factors play a large role in the CCT variation seen between ethnic groups. The genes responsible however, remain largely unknown, although our data from the pigment analysis may offer some clues.

Upon segregating the mouse strains into either pigmented or albino groups, the albino animals were found to have a significantly thicker CCT. Of the seven albino strains measured, six had greater mean CCT measurements than the C57BL/6J mouse, which was thickest pigmented strain. This result was consistent with the human data and supported the hypothesis that darker skin or coat pigmentation is associated with a thinner CCT. The use of a murine model can also alleviate many of the problems faced in human studies and can potentially offer a more accurate representation of the influence that pigment has on CCT. Our experimental design allowed us to control for variables that may influence data from human studies, including the type of pachymeter, pachymeter operator, sex and age. The impact of environmental factors was also limited, with the mice kept in the same living conditions and on the same diet. The use of inbred mice also reduced any intra-strain variation that could result from genetic differences. If the association between CCT and coat pigmentation is a genuine biological interaction, then it is plausible that genes involved in pigment biosynthesis are also involved in the development of the cornea. To date, some 368 mouse colour loci have been identified, of which 159 are cloned genes, ensuring there are numerous candidate genes that could be involved in CCT determination [47].

To identify what genes may be responsible for this variability in CCT, genotyping information was obtained for specific mutations within five known mouse pigment genes (http://www.jax.org/). The goal of this analysis was to ascertain if the melanin biosynthesis pathway was potentially linked to CCT determination. The genes investigated were *a*, *Myo5a*, *Oca2*, *Tyr* and *Tyrp1.* Mutations in these genes have varying effects on pigment expression, whilst the combination of alleles is also important to the final phenotype of the mouse. Our results indicated that the *a*, *Myo5a^d^*, *Tyr^c^* and *Tyrp1^b^* alleles were all significantly associated with a difference in CCT when compared to the wild type alleles. In particular, the *a*, *Myo5a^d^* and *Tyrp1^b^* alleles all showed a highly significant association with a thinner CCT. What potential role these genes may play in the determination of CCT however, is unclear. The function of the nonagouti protein is to act as a ligand for the melanocortin-1 receptor and regulate the ratio of eumelanin to pheomelanin production, whilst Myo5a is involved in cellular motility and mutations are believed to disrupt pigment biosynthesis through abnormal trafficking of melanosomes within melanocytes [48]. Tyr is the rate-limiting enzyme in the bioysnthesis of melanin and catalyses the initial step, the conversion of tyrosine to DOPAquinone, whereas tyrp1 is known to catalyse the formation of an intermediate in the eumelanin synthesis pathway and to stabilise the tyrosinase protein [49]–[51]. None of these protein functions appear to have any obvious involvement in corneal development, although this probably reflects the novelty of the association between pigment and CCT. It is also unclear as to whether these genes are acting independently or whether there is a combined effect on overall pigmentation levels. Other pigment-related genes in addition to the five investigated in this study may also be contributing to the association with CCT. Further work is needed to verify the relationship between *a*, *Myo5a^d^*, *Tyr^c^* and *Tyrp1^b^* and CCT, including measurement in additional mouse strains, expression and functional analysis and potentially, examination of these genes in human cohorts.

The concept that genes associated with pigment regulation are also involved in the development of a transparent tissue such as the cornea is extremely novel. Previous data from human studies has confirmed the presence of melanocytes and their product melanin at the corneo-scleral junction of adults, although the function of a pigmented limbus remains unknown [52]. Similar observations have been made in an avian model, where melanocytes are present in the corneal limbus of day 13 embryos, indicating that expression of pigment genes is evident in the tissue directly adjacent to the developing cornea [53]. Mutations in *TYR, P, TYRP1* and *SLC45A2* are also known to be associated with the significant ocular abnormalities seen in OCA patients [42], while a study by Libby *et al* has demonstrated that *Tyr* mutations can increase the severity of anterior segment dysgenesis in the mouse [54]. These abnormalities indicate that genes associated with pigmentation pathways are intimately involved in the development of ocular structures and this may include the cornea. Given that a healthy adult cornea is transparent, regulation of its growth by pigment associated genes would suggest that these genes may play a more significant role in mammalian development than what is currently understood. Several recent genome-wide association studies have identified *COL5A1*, *COL8A2*, *AKAP13, AVGR8, FOX01* and *ZNF469* as determinants of normal CCT variation [19]–[21]. However, to our knowledge there is no evidence that any of these genes are involved in the regulation of human pigmentation. This could be due to the fact that these studies were only performed on ethnically homogenous populations, within which variation in pigmentation levels is limited.

The findings presented in this paper provide further evidence that CCT is a genetically determined trait. As seen with the ethnic variation in humans, significant strain differences in CCT are evident in the mouse. Furthermore, the data presented on the correlation of pigment with CCT in both the normal and mutant strains indicates that genes involved in the pigment pathways may also be candidate CCT genes. The benefits of characterising these genes lie beyond the mere identification of quantitative trait loci, as they may offer insights into other developmental pathways that are associated with pigmentation.

## Supporting Information

Table S1 **Studies included in meta-analysis of human CCT measurements.**
(DOC)
