# Supplementary material for: Ethnic and Mouse Strain Differences in Central Corneal Thickness and Association with Pigmentation Phenotype
Source: PLoS One. 2011 Aug 10;6(8):e22103. doi: 10.1371/journal.pone.0022103 (PMC3154201; doi:10.1371/journal.pone.0022103)
Supplement: Table S1 — Studies included in meta-analysis of human CCT measurements. (DOC) [file pone.0022103.s001.doc]

| **Ethnic Group** | **Country of Study** | **Number of Participants** | **Mean CCT±SD (µm)** | **Glaucoma Included** | **Reference** |
| --- | --- | --- | --- | --- | --- |
| Australian Aboriginal | Australia | 189 | 514.9±30.5 | No | Durkin *et al* [1] |
| Australian Aboriginal | Australia | 91 | 509±33.5 | No | Landers *et al* [2] |
| African Migrant | Barbados | 1064 | 529.8±37.7 | Yes | Nemesure *et al* [3] |
| African Migrant | Canada | 32 | 529.7±30** | No | Dohadwala *et al* [4] |
| African Migrant | Israel | 121 | 518.9±31.5 | No | Lifshitz *et al* [5] |
| African Migrant | Peurto Rico | 98 | 542±32 | Yes | Graeber *et al* [6] |
| African Migrant | USA | 26 | 524.8±38.4 | No | Aghaian *et al* [7] |
| African Migrant | USA | 26 | 533.8±33.9 | No | La Rosa *et al* [8] |
| African Migrant | USA | 393 | 533.8±33.7 | No | Racette *et al* [9] |
| African Migrant | USA | 18 | 533±37.8 | No | Semes *et al* [10] |
| African Migrant | USA | 58* | 535.8±33.4 | No | Shimmyo *et al* [11] |
| African Migrant | USA | 33 | 528.5±33.2 | No | Torres *et al* [12] |
| African Migrant | USA | 36 | 530±34.2 | ? | Yo *et al* [13] |
| African Native | Cameroon | 485 | 528.7±35.9 | No | Eballe *et al* [14] |
| African Native | Ethiopia | 300 | 518.7±32.9 | No | Gelaw *et al* [15] |
| African Native | Ghana | 155 | 525.3±33.5 | ? | Kim *et al* [16] |
| African Native | Nigeria | 49 | 551.6±44.5 | No | Iyamu *et al* [17] |
| African Native | Nigeria | 34 | 535±38 | No | Mercieca *et al* [18] |
| African Native | Uganda | 297 | 517.3±37 | ? | Current Study |
| Caucasian European | Austria | 30 | 552±31.7 | ? | Lackner *et al* [19] |
| Caucasian European | Croatia (Korcula) | 849 | 555.6±36 | ? | Vitart *et al* [20] |
| Caucasian European | Croatia (Split) | 349 | 561±36.3 | ? | Vitart *et al* [21] |
| Caucasian European | Croatia (Vis) | 596 | 561.2±34.6 | ? | Vitart *et al* [20] |
| Caucasian European | Greece | 57 | 547.4±33.1 | No | Kitsos *et al* [22] |
| Caucasian European | Hungary | 20 | 559±30.7 | No | Schneider *et al* [23] |

**Table S1.** Studies included in meta-analysis of human CCT measurements

* Study originally quoted total number of eyes measured

** Standard deviation calculated from standard error

| **Ethnic Group** | **Country of Study** | **Number of Participants** | **Mean CCT±SD (µm)** | **Glaucoma Included** | **Reference** |
| --- | --- | --- | --- | --- | --- |
| Caucasian European | Netherlands | 352 | 537.4±33.8** | No | Wolfs *et al* [24] |
| Caucasian European | Scotland | 475 | 536±33.4 | ? | Vitart *et al* [21] |
| Caucasian European | Spain | 100 | 557.5±15 | ? | Sanchis-Gimeno *et al* [25] |
| Caucasian European | Switzerland | 18 | 552±35 | No | Copt *et al* [26] |
| Caucasian European | England | 983 | 544.1±36.5 | No | Hawker *et al* [27] |
| Caucasian European | England | 1759 | 545.8±34 | Yes | Lu *et al* [28] |
| Caucasian Migrant | Australia | 115 | 544.7±31.9 | No | Durkin *et al* [1] |
| Caucasian Migrant | Australia | 84 | 542±32 | No | Landers *et al* [2] |
| Caucasian Migrant | Australia | 1714 | 544.3±35 | ? | Lu *et al* [28] |
| Caucasian Migrant | Australia | 956 | 539.7±32.8 | Yes | This Study |
| Caucasian Migrant | Barbados | 25 | 545.2±45.7 | Yes | Nemesure *et al* [3] |
| Caucasian Migrant | Canada | 227 | 552.5±34.7** | No | Dohadwala *et al* [4] |
| Caucasian Migrant | Peurto Rico | 361 | 542±32 | Yes | Graeber *et al* [6] |
| Caucasian Migrant | USA | 36 | 562.8±31.1 | No | Aghaian *et al* [7] |
| Caucasian Migrant | USA | 51 | 555.9±33.2 | No | La Rosa *et al* [8] |
| Caucasian Migrant | USA | 101 | 554±34 | ? | Phillips *et al* [29] |
| Caucasian Migrant | USA | 367 | 551.9±36.8 | No | Racette *et al* *[9]* |
| Caucasian Migrant | USA | 38 | 562±31 | No | Realini *et al* [30] |
| Caucasian Migrant | USA | 48 | 556.1±38.8 | No | Semes *et al* [10] |
| Caucasian Migrant | USA | 733* | 552.6±34.5 | No | Shimmyo *et al* [11] |
| Caucasian Migrant | USA | 46 | 551.9±28.3 | No | Torres *et al* [12] |
| Caucasian Migrant | USA | 138 | 545±33.9 | ? | Yo *et al* [13] |
| East Asian | China | 1669 | 548.6±34.3 | No | Li *et al* [31] |
| East Asian | Hong Kong | 151 | 575±32 | No | Cho *et al* [32] |
| East Asian | Hong Kong | 240 | 551.7±30.6 | No | Lam *et al* [33] |
| East Asian | Hong Kong | 125 | 560.3±22.7 | No | Lam *et al* [34] |

**Table S1.** *Continued*

* Study originally quoted total number of eyes measured

** Standard deviation calculated from standard error

| **Ethnic Group** | **Country of Study** | **Number of Participants** | **Mean CCT±SD (µm)** | **Glaucoma Included** | **Reference** |
| --- | --- | --- | --- | --- | --- |
| East Asian | Hong Kong | 50 | 543±33 | No | Leung *et al* [35] |
| East Asian | Hong Kong | 39 | 555.1±35.3 | No | Wong *et al* [36] |
| East Asian | Japan | 50 | 552±36 | No | Wu *et al* [37] |
| East Asian | Korea | 205 | 535.8±36 | No | Kim *et al* [38] |
| East Asian | Korea | 224 | 553.6±39.6 | No | Lee *et al* [39] |
| East Asian | Taiwan | 500 | 554±29 | No | Chen *et al* [40] |
| East Asian | Taiwan | 56 | 567±43 | No | Ko *et al* [41] |
| East Asian | USA | 41 | 569.5±31.8 | No | Aghaian *et al* [7] |
| East Asian | USA | 38 | 538.5±29.6 | No | Aghaian *et al* [7] |
| East Asian | USA | 136 | 554.8±38.8 | No | Pekmezci *et al* [42] |
| Hispanic | USA | 27 | 563.6±29.1 | No | Aghaian *et al* [7] |
| Hispanic | USA | 104 | 541.8±34.5 | No | Erickson *et al* [43] |
| Hispanic | USA | 1699 | 546.9±33.5 | Yes | Hahn *et al* [44] |
| Hispanic | USA | 102* | 551.1±35.5 | No | Shimmyo *et al* [11] |
| Hispanic | USA | 139 | 542±34.6 | ? | Yo *et al* [13] |
| South Asian | India | 2532 | 520.7±33.4 | No | Vijaya *et al* [45] |
| South Asian | India | 532 | 532.2±34 | No | Kohli *et al* [46] |
| South Asian | India | 615 | 519.9±33.4 | ? | Kunert *et al* [47] |
| South Asian | India | 46 | 541.8±30.6 | No | Ladi *et al* [48] |
| South Asian | India | 4612 | 514±33 | ? | Nangia *et al* [49] |
| South Asian | Pakistan | 100 | 531.1±33.4 | No | Channa *et al* [50] |
| South East Asian | Burma | 1909 | 521.9±33.3 | No | Casson *et al* [51] |
| South East Asian | Thailand | 50 | 554.4±27.5 | ? | Chaidaroon *et al* [52] |
| South East Asian | Thailand | 467 | 535.2±29.9 | ? | Lekskul *et al* [53] |
| South East Asian | USA | 33 | 559±24.9 | No | Aghaian *et al* [7] |

**Table S1.** *Continued*

* Study originally quoted total number of eyes measured

**REFERENCES**

1. Durkin SR, Tan EW, Casson RJ, Selva D, Newland HS (2007) Central corneal thickness among Aboriginal people attending eye clinics in remote South Australia. Clin Experiment Ophthalmol 35: 728-732.

2. Landers JA, Billing KJ, Mills RA, Henderson TR, Craig JE (2007) Central corneal thickness of indigenous Australians within Central Australia. Am J Ophthalmol 143: 360-362.

3. Nemesure B, Wu SY, Hennis A, Leske MC (2003) Corneal thickness and intraocular pressure in the Barbados eye studies. Arch Ophthalmol 121: 240-244.

4. Dohadwala AA, Munger R, Damji KF (1998) Positive correlation between Tono-Pen intraocular pressure and central corneal thickness. Ophthalmology 105: 1849-1854.

5. Lifshitz T, Levy J, Rosen S, Belfair N, Levinger S (2006) Central corneal thickness and its relationship to the patient's origin. Eye 20: 460-465.

6. Graeber CP, Torres MB, Shields MB (2008) Central corneal thickness in a Puerto Rican population. J Glaucoma 17: 356-360.

7. Aghaian E, Choe JE, Lin S, Stamper RL (2004) Central corneal thickness of Caucasians, Chinese, Hispanics, Filipinos, African Americans, and Japanese in a glaucoma clinic. Ophthalmology 111: 2211-2219.

8. La Rosa FA, Gross RL, Orengo-Nania S (2001) Central corneal thickness of Caucasians and African Americans in glaucomatous and nonglaucomatous populations. Arch Ophthalmol 119: 23-27.

9. Racette L, Liebmann JM, Girkin CA, Zangwill LM, Jain S, et al. (2010) African Descent and Glaucoma Evaluation Study (ADAGES): III. Ancestry differences in visual function in healthy eyes. Arch Ophthalmol 128: 551-559.

10. Semes L, Shaikh A, McGwin G, Bartlett JD (2006) The relationship among race, iris color, central corneal thickness, and intraocular pressure. Optom Vis Sci 83: 512-515.

11. Shimmyo M, Ross AJ, Moy A, Mostafavi R (2003) Intraocular pressure, Goldmann applanation tension, corneal thickness, and corneal curvature in Caucasians, Asians, Hispanics, and African Americans. Am J Ophthalmol 136: 603-613.

12. Torres RJ, Jones E, Edmunds B, Becker T, Cioffi GA, et al. (2008) Central Corneal Thickness in Northwestern American Indians/Alaskan Natives and Comparison with White and African-American Persons. Am J Ophthalmol.

13. Yo C, Ariyasu RG (2005) Racial differences in central corneal thickness and refraction among refractive surgery candidates. J Refract Surg 21: 194-197.

14. Eballe AO, Koki G, Ellong A, Owono D, Epee E, et al. (2010) Central corneal thickness and intraocular pressure in the Cameroonian nonglaucomatous population. Clin Ophthalmol 4: 717-724.

15. Gelaw Y, Kollmann M, Irungu NM, Ilako DR (2010) The Influence of Central Corneal Thickness on Intraocular Pressure Measured by Goldmann Applanation Tonometry Among Selected Ethiopian Communities. J Glaucoma.

16. Kim HY, Budenz DL, Lee PS, Feuer WJ, Barton K (2008) Comparison of central corneal thickness using anterior segment optical coherence tomography vs ultrasound pachymetry. Am J Ophthalmol 145: 228-232.

17. Iyamu E, Ituah I (2008) The relationship between central corneal thickness and intraocular pressure: a comparative study of normals and glaucoma subjects. Afr J Med Med Sci 37: 345-353.

18. Mercieca K, Odogu V, Fiebai B, Arowolo O, Chukwuka F (2007) Comparing central corneal thickness in a sub-Saharan cohort to African Americans and Afro-Caribbeans. Cornea 26: 557-560.

19. Lackner B, Schmidinger G, Pieh S, Funovics MA, Skorpik C (2005) Repeatability and reproducibility of central corneal thickness measurement with Pentacam, Orbscan, and ultrasound. Optom Vis Sci 82: 892-899.

20. Vitart V, Bencic G, Hayward C, Herman JS, Huffman J, et al. (2010) Heritabilities of ocular biometrical traits in two croatian isolates with extended pedigrees. Invest Ophthalmol Vis Sci 51: 737-743.

21. Vitart V, Bencic G, Hayward C, Herman JS, Huffman J, et al. (2010) New loci associated with central cornea thickness include COL5A1, AKAP13 and AVGR8. Hum Mol Genet.

22. Kitsos G, Gartzios C, Asproudis I, Bagli E (2009) Central corneal thickness in subjects with glaucoma and in normal individuals (with or without pseudoexfoliation syndrome). Clin Ophthalmol 3: 537-542.

23. Schneider M, Borgulya G, Seres A, Nagy Z, Nemeth J (2009) Central corneal thickness measurements with optical coherence tomography and ultrasound pachymetry in healthy subjects and in patients after photorefractive keratectomy. Eur J Ophthalmol 19: 180-187.

24. Wolfs RC, Klaver CC, Vingerling JR, Grobbee DE, Hofman A, et al. (1997) Distribution of central corneal thickness and its association with intraocular pressure: The Rotterdam Study. Am J Ophthalmol 123: 767-772.

25. Sanchis-Gimeno JA, Lleo-Perez A, Alonso L, Rahhal MS (2004) Caucasian emmetropic aged subjects have reduced corneal thickness values: emmetropia, CCT and age. Int Ophthalmol 25: 243-246.

26. Copt RP, Thomas R, Mermoud A (1999) Corneal thickness in ocular hypertension, primary open-angle glaucoma, and normal tension glaucoma. Arch Ophthalmol 117: 14-16.

27. Hawker MJ, Edmunds MR, Vernon SA, Hillman JG, Macnab HK (2007) The relationship between central corneal thickness and the optic disc in an elderly population: the Bridlington Eye Assessment Project. Eye.

28. Lu Y, Dimasi DP, Hysi PG, Hewitt AW, Burdon KP, et al. (2010) Common genetic variants near the Brittle Cornea Syndrome locus ZNF469 influence the blinding disease risk factor central corneal thickness. PLoS Genet 6: e1000947.

29. Phillips LJ, Cakanac CJ, Eger MW, Lilly ME (2003) Central corneal thickness and measured IOP: a clinical study. Optometry 74: 218-225.

30. Realini T, Weinreb RN, Hobbs G (2009) Correlation of intraocular pressure measured with goldmann and dynamic contour tonometry in normal and glaucomatous eyes. J Glaucoma 18: 119-123.

31. Li P, Hu Y, Xu Q, Zhang G, Mai C (2006) Central corneal thickness in adult Chinese. J Huazhong Univ Sci Technolog Med Sci 26: 141-144.

32. Cho P, Lam C (1999) Factors affecting the central corneal thickness of Hong Kong-Chinese. Curr Eye Res 18: 368-374.

33. Lam AK, Douthwaite WA (1998) The corneal-thickness profile in Hong Kong Chinese. Cornea 17: 384-388.

34. Lam A, Chen D, Chiu R, Chui WS (2007) Comparison of IOP measurements between ORA and GAT in normal Chinese. Optom Vis Sci 84: 909-914.

35. Leung DY, Lam DK, Yeung BY, Lam DS (2006) Comparison between central corneal thickness measurements by ultrasound pachymetry and optical coherence tomography. Clin Experiment Ophthalmol 34: 751-754.

36. Wong AC, Wong CC, Yuen NS, Hui SP (2002) Correlational study of central corneal thickness measurements on Hong Kong Chinese using optical coherence tomography, Orbscan and ultrasound pachymetry. Eye 16: 715-721.

37. Wu LL, Suzuki Y, Ideta R, Araie M (2000) Central corneal thickness of normal tension glaucoma patients in Japan. Jpn J Ophthalmol 44: 643-647.

38. Kim JM, Park KH, Kim SH, Kang JH, Cho SW (2010) The relationship between the cornea and the optic disc. Eye (Lond).

39. Lee ES, Kim CY, Ha SJ, Seong GJ, Hong YJ (2007) Central corneal thickness of Korean patients with glaucoma. Ophthalmology 114: 927-930.

40. Chen MJ, Liu YT, Tsai CC, Chen YC, Chou CK, et al. (2009) Relationship between central corneal thickness, refractive error, corneal curvature, anterior chamber depth and axial length. J Chin Med Assoc 72: 133-137.

41. Ko YC, Liu CJ, Hsu WM (2005) Varying effects of corneal thickness on intraocular pressure measurements with different tonometers. Eye (Lond) 19: 327-332.

42. Pekmezci M, Vo B, Lim AK, Hirabayashi DR, Tanaka GH, et al. (2009) The characteristics of glaucoma in Japanese Americans. Arch Ophthalmol 127: 167-171.

43. Erickson DH, Goodwin D, Anderson C, Hayes JR (2010) Ocular pulse amplitude and associated glaucomatous risk factors in a healthy Hispanic population. Optometry 81: 408-413.

44. Hahn S, Azen S, Ying-Lai M, Varma R (2003) Central corneal thickness in Latinos. Invest Ophthalmol Vis Sci 44: 1508-1512.

45. Vijaya L, George R, Baskaran M, Arvind H, Raju P, et al. (2008) Prevalence of primary open-angle glaucoma in an urban south Indian population and comparison with a rural population. The Chennai Glaucoma Study. Ophthalmology 115: 648-654 e641.

46. Kohli PG, Randhawa BK, Singh KD, Randhawa GS, Kohli AK (2010) Relation between central corneal thickness and intraocular pressure in Punjabi population. J Med Eng Technol 34: 1-6.

47. Kunert KS, Bhartiya P, Tandon R, Dada T, Christian H, et al. (2003) Central corneal thickness in Indian patients undergoing LASIK for myopia. J Refract Surg 19: 378-379.

48. Ladi JS, Shah NA (2010) Comparison of central corneal thickness measurements with the Galilei dual Scheimpflug analyzer and ultrasound pachymetry. Indian J Ophthalmol 58: 385-388.

49. Nangia V, Jonas JB, Sinha A, Matin A, Kulkarni M (2010) Central corneal thickness and its association with ocular and general parameters in Indians: the Central India Eye and Medical Study. Ophthalmology 117: 705-710.

50. Channa R, Mir F, Shah MN, Ali A, Ahmad K (2009) Central corneal thickness of Pakistani adults. J Pak Med Assoc 59: 225-228.

51. Casson RJ, Abraham LM, Newland HS, Muecke J, Sullivan T, et al. (2008) Corneal thickness and intraocular pressure in a nonglaucomatous Burmese population: the Meiktila Eye Study. Arch Ophthalmol 126: 981-985.

52. Chaidaroon W (2003) The comparison of corneal thickness measurement: ultrasound versus optical methods. J Med Assoc Thai 86: 462-466.

53. Lekskul M, Aimpun P, Nawanopparatskul B, Bumrungsawat S, Trakulmungkijkarn T, et al. (2005) The correlations between Central Corneal Thickness and age, gender, intraocular pressure and refractive error of aged 12-60 years old in rural Thai community. J Med Assoc Thai 88 Suppl 3: S175-179.
